# Urine High-Sensitivity Troponin I Predict Incident Cardiovascular Events in Patients with Diabetes Mellitus

**DOI:** 10.3390/jcm9123917

**Published:** 2020-12-02

**Authors:** Ju-Yi Chen, Shuenn-Yuh Lee, Yi-Heng Li, Chia-Yu Lin, Meng-Dar Shieh, Ding-Siang Ciou

**Affiliations:** 1 Division of Cardiology, Department of Internal Medicine, National Cheng Kung University Hospital, College of Medicine, National Cheng Kung University, Tainan 704, Taiwan; heng@mail.ncku.edu.tw; 2 Department of Electrical Engineering, National Cheng-Kung University, Tainan 704, Taiwan; ieesyl@mail.ncku.edu.tw (S.-Y.L.); gary4842002@yahoo.com.tw (D.-S.C.); 3 Department of Chemical Engineering, National Cheng-Kung University, Tainan 704, Taiwan; cyl44@mail.ncku.edu.tw; 4 Department of Industrial Design, National Cheng-Kung University, Tainan 704, Taiwan; mdshieh2019@gmail.com

**Keywords:** urine, troponin, diabetes mellitus, incident cardiovascular events

## Abstract

In patients with diabetes mellitus (DM), incident cardiovascular (CV) events are associated with poor long-term outcomes. Serum high-sensitivity troponin I (hs-TnI) is widely used to diagnose and predict outcomes in patients with acute coronary syndrome, however, few studies have investigated the accuracy of urine hs-TnI as a predictor for incident CV events in patients with DM. The enrolled participants included patients with DM. Fresh urine hs-TnI levels were measured. Medical records of enrolled patients were used to determine the number of incident CV events prospectively for 3 months. The study cohort comprised 378 participants. We observed significantly higher levels of urine hs-TnI in those with than without subsequent incident CV events. The multivariate logistic regression analysis using different models consistently showed that urine hs-TnI > 4.10 pg/mL was an independent factor predictive of incident CV events. The ROC-AUC analysis revealed that the optimal cutoff value for urine hs-TnI for predicting incident CV events was 1.55 pg/mL and the area was 0.611 (*p* = 0.027). A single measurement of urinary hs-TnI, collected easily and non-invasively, may be an acceptable biomarker for predicting subsequent incident CV events in patients with DM.

**Highlights**

- Serum high-sensitivity troponin I (hs-TnI) is a well-established acute coronary syndrome biomarker used for diagnosis and to predict prognosis.
- We demonstrate that a single measurement of hs-TnI in fresh urine could be an acceptable marker for predicting incident cardiovascular events in patients with diabetes mellitus.
- A single measurement of urinary hs-TnI may be an acceptable biomarker for predicting incident cardiovascular events in patients with diabetes mellitus.

## 1. Introduction

Diabetes mellitus (DM), a well-established high-risk disease, is significantly associated with subsequent microvascular events, including neuropathy, retinopathy and nephropathy, and macrovascular events, including acute coronary syndrome, stroke, and peripheral arterial disease [1]. Although co-morbidities and clinical characteristics are useful for stratifying and predicting the risk of ischemic and heart failure events in DM patients, the use of cardiovascular biomarkers to better classify the risk will improve patient care [2]. Serum high-sensitivity cardiac troponin I (hs-TnI) is a sensitive biomarker for the diagnosis of acute coronary syndrome (ACS) [3] and is associated with adverse cardiovascular outcomes in patients with stable coronary atherosclerotic disease [4] and in those with type 2 DM [5,6]. A recent study also demonstrated that among patients without ACS, serum hs-TnI is a stronger predictor of cardiovascular outcomes, including major adverse cardiovascular events and mortality, in those with than without chronic kidney disease (CKD) [7].

Unfortunately, the blood test for hs-TnI is invasive, skill-dependent, and inconvenient for use in outpatient departments or as a screening tool. A recently-developed hs-TnI immunoassay has been shown to detect hs-TnI in urine at concentrations 10,000-fold than that in plasma [8]. Due to its simplicity and cost-effectiveness, the urinary hs-TnI measurement is a promising new tool for diagnosing and monitoring hypertensive patients [9]. The predictive value of urinary hs-TnI in patients with DM has not been investigated. This study aims to determine whether urinary hs-TnI is independently associated with subsequent incident cardiovascular (CV) events in patients with DM.

## 2. Methods

### 2.1. Patient Cohort

This prospective case-control study enrolled all patients treated in the cardiovascular outpatient department of National Cheng Kung University Hospital from December 2018 to December 2019 who had a diagnosis of type 2 DM. Patients with end-stage renal disease or CV events within one month before enrolment were excluded. All patients received standard therapy for DM based on current guidelines [10]. Medical histories, including co-morbid diseases and co-medications, were reviewed. Type 2 DM was diagnosed if the fasting plasma glucose concentration was >126 mg/dL or hemoglobin A1C level was >6.5% on two separate occasions or if the patient was being treated with insulin or oral hypoglycemic agents. Hypertension was diagnosed if blood pressure was >140/90 mm Hg on three separate occasions or if the patient was being treated with an antihypertensive medication. Hypercholesterolemia was defined as a total serum cholesterol concentration ≥ 200 mg/dL or the use of lipid-lowering therapy. Coronary artery disease was defined as a positive history of acute coronary syndrome, percutaneous coronary intervention with stents, or positive stress test results. Chronic kidney disease was defined as an estimated glomerular filtration rate < 60 mL/min/1.73 m^2^ for 3 months. Heart failure was defined as typical manifestations with either left ventricular ejection fraction > 50% or <50%. The primary endpoint was the number of incident CV events for cardiovascular reasons within 3 months after enrollment. The medical records of enrolled patients were reviewed for information regarding incident CV events (heart failure or acute coronary syndrome, including unstable angina or acute myocardial infarction, CV death, and all-cause mortality) after 3 months of sampling blood and urine. During enrollment, blood and fresh urine samples were collected, and the blood pressure, heart rate, and body mass index (BMI) were measured. This study was approved by the ethics committee of National Cheng Kung University Hospital (A-ER-106-421) and was conducted according to the guidelines of the International Conference on Harmonization for Good Clinical Practice. All patients provided a written informed consent before enrollment. 

### 2.2. Laboratory Examinations

Blood and fresh urine samples were collected. Fresh, unfrozen samples were sent to the chemistry laboratory at National Cheng Kung University. Urine samples were collected during outpatient department visits from 9:00 a.m.–12:00 p.m. or 2:00–5:00 p.m. Serum creatinine levels were used to assess the estimated glomerular filtration rate (eGFR) calculated with the Modified Diet in Renal Disease Equation. The urine creatinine and albumin concentrations were determined using creatinine FS (DiaSys Diagnostic Systems GmbH, Holzheim, Germany) and the K-ASSAY microalbumin assay (Kamiya Biomedical Company, Seattle, WA, USA), as well as an automatic biochemical analyzer (TBA-25 FR, Toshiba Medical Systems, Tokyo, Japan). The measurement range was 0.20–15.00 mg/dL for creatinine and 0.20–30.00 mg/dL for albumin. The hs-TnI concentration was measured using a miniVIDAS analyzer (BioMérieux SA, Mercy I’Etoile, France) with a coefficient of variation ≤ 10%. The detectable range of hs-TnI was 0.75–40,000 pg/mL. For values exceeding this range, samples were diluted with normal saline and remeasured. The urine sample (200 μL) was pipetted onto a VIDAS TNHS test strip (BioMérieux SA, Mercy I’Etoile, France), which then was inserted into the miniVIDAS analyzer. The assay combines a one-step enzyme immunoassay sandwich method with fluorescence detection (ELFA). The solid phase receptacle (SPR) serves as the solid phase as well as the pipetting device. The interior of the SPR is coated with a mouse monoclonal anti-cardiac troponin I immunoglobulin. The sample is transferred into a well containing alkaline phosphatase-labeled anti-cardiac troponin antibodies (conjugate). The sample/conjugate mixture is cycled in and out of the SPR several times, promoting the binding of the troponin I to the immunoglobulin fixed to the wall of the SPR to form a sandwich. The Troponin I assay shows high specificity towards troponin I against cross-reactive compounds (skeletal troponin I, cardiac troponin C, cardiac troponin T, and skeletal troponin T). The cross-reactivity (%) towards skeletal troponin I, cardiac troponin C, cardiac troponin T, and skeletal troponin T is less than 0.1%. Detailed information is available on the product data sheet (VIDAS High Sensitive Troponin I, REF 415386).

### 2.3. Statistical Analysis

All data were analyzed using the SPSS statistical package version 23.0 (SPSS Inc. Chicago, IL, USA). Data are expressed as the mean ± standard deviation. Continuous variables were compared using the Student’s t-test (if the variable was a normal distribution) or Mann-Whitney U-test (if the variable was not a normal distribution). Categorical variables were compared using the Chi-Square or Fisher’s exact test. All tests were two-tailed, and *p* < 0.05 was considered statistically significant. The multivariate logistic regression analysis was conducted in different modes and adjusted for different confounders (for *p* < 0.05 in the univariate analysis) to identify independent factors associated with subsequent incident CV events. Sine urine hs-TnI values did not exhibit a normal distribution, the median value (4.10 pg/mL) was used as a cutoff in the multivariate analysis. The Kaplan-Meier curve was used for validation of our analysis. The receiver-operating characteristic (ROC) area under the curve (AUC) for urine hs-TnI and the associated 95% confidence intervals were investigated for association with subsequent incident CV events. The cutoff values were chosen based on the results of the ROC curve analysis and used to analyze the associated values of urine hs-TnI in detecting end points. 

## 3. Results

### 3.1. Patient Baseline Characteristics 

Baseline characteristics of the 378 enrolled patients are shown in Table 1. Patients were predominantly male (65.6%). The most common co-morbidity in this study population was hypertension (275 patients; 72.8%). Other co-morbidities included heart failure with reduced left ventricular ejection fraction (*n* = 66; 17.5%), coronary artery disease (*n* = 166; 43.9%), atrial fibrillation (24.9%), and CKD (29.6%). Of the 378 patients, 143 (37.8%) had microalbuminuria (urine albumin-creatinine ratio (UACR) > 30 and <300) and 40 (10.6%) had macroalbuminuria (UACR > 300). Most of the patients had well-controlled systolic and diastolic blood pressures. Most of the patients received guideline-derived medical therapy: More than 75% of the patients were taking *renin-angiotensin-aldosterone system* (RAAS) inhibitors, 52.6% were taking biguanide, and 14.8% were taking a sodium glucose co-transporters 2 (SGLT_2_) inhibitor. No patients took *glucagon-like peptide 1* receptor *agonist.*

### 3.2. Parameter Comparison between Patients with DM with or without Subsequent Incident Cardiovascular Events within 3 Months 

There were 37 (9.8%) patients with incident CV events: None for CV death or all-cause mortality, 21 for heart failure, 12 for unstable angina, and 4 for acute myocardial infarction. More patients with subsequent incident CV events had a history of heart failure with reduced ejection fraction than did those without (14/37 [37.8%] vs. 52/341 [15.2%]; *p* = 0.001) (Table 1). No significant difference was observed between patients with and without previous incident CV events with respect to age, sex, BMI, blood pressure, heart rate, other co-morbidities, or co-medications (Table 1), except for significantly lower eGFR in patients with subsequent incident CV events (62.1 ± 24.1 vs. 70.8 ± 22.8 mL/min/1.73 m^2^, *p* = 0.029). No significant difference was observed in the presence of microalbuminuria (8.0% vs. 13.3%, *p* = 0.100) or macroalbuminuria (9.5% vs. 15.0%, *p* = 0.271). Two hundred and thirty patients (60.8%) had a urine hs-TnI level of 0.75 pg/mL due to the limit of detection. The mean urine hs-TnI was 5.98 pg/mL in 148 patients, with a median value of 4.10 pg/mL. Since urine hs-TnI values was not normally distributed, we used the median value of 4.10 pg/mL as the cutoff for grouping. More patients with subsequent incident CV events had urine hs-TnI > 4.10 pg/mL than did those without (40.5% vs. 17.6%, *p* = 0.001). 

### 3.3. Multivariate Logistic Regression Analysis for Independent Predictors of Subsequent Incident CV Events

**Figure 1:**
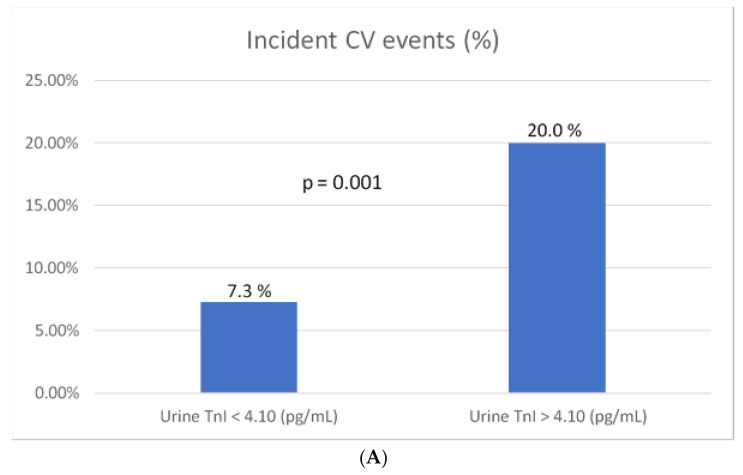

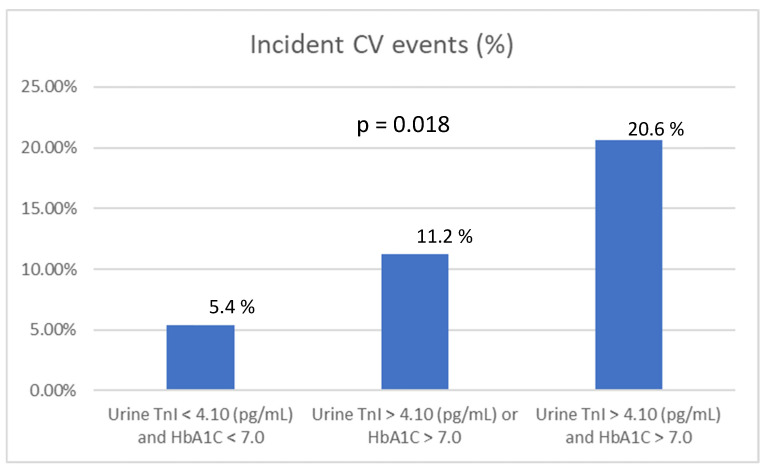
Frequency of subsequent incident cardiovascular (CV) events according to the urine high-sensitivity troponin I (hs-TnI) concentration and HbA1C level. (**A**) The percentage of patients with subsequent incident CV events was significantly higher in those with a hs-TnI concentration > 4.10 pg/mL; (**B**) the percentage of patients with subsequent incident CV events was significantly higher in those with a hs-TnI concentration > 4.10 pg/mL and HbA1C > 7.0 %.

**Figure 2:**
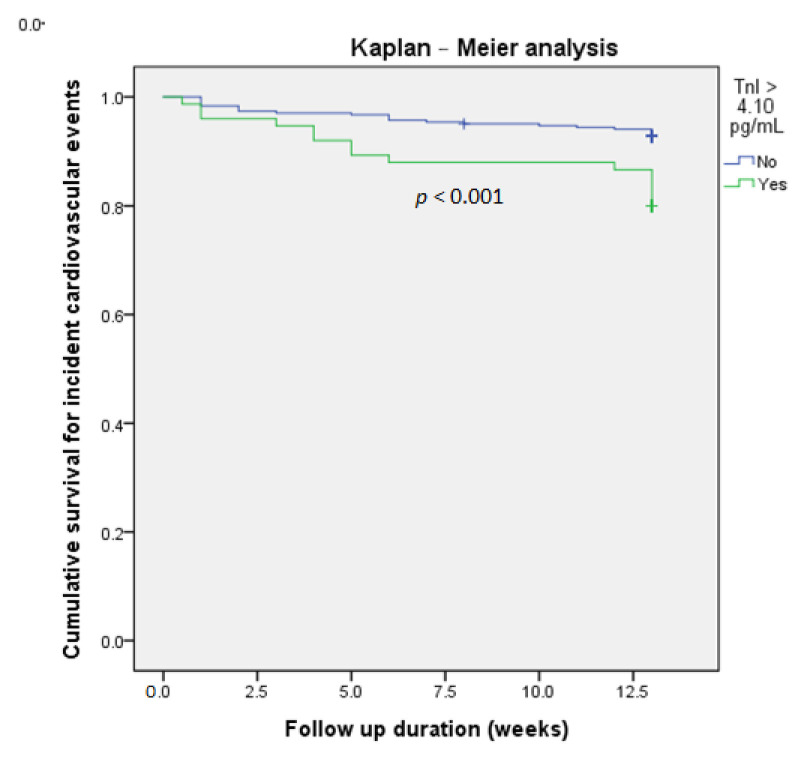
Kaplan-Meier curve analysis of fresh urine hs-TnI concentration > 4.10 pg/mL vs. cumulative survival for incident cardiovascular events in patients with diabetes mellitus (DM).

Both age and sex are essential confounding factors which were included in all models. Other covariates with *p* < 0.05, including eGFR and past history of heart failure with reduced ejection fraction, were included (Table 1). The multivariate logistic regression analysis was conducted using different models to identify independent factors predictive of subsequent incident CV events. Urine hs-TnI > 4.10 pg/mL remained an independent factor, even after adjusting for hemoglobin A1C, an important index of DM control (odds ratio, 3.043; CI, 1.448–6.392; *p* = 0.003) (Table 2). Patients with urine hs-TnI > 4.10 pg/mL had a significantly higher risk of subsequent incident CV events (*p* = 0.001) (Figure 1A). We further divided the study population into three groups based on the median value of urine hs-TnI (4.10 pg/mL) and hemoglobin A1C levels (7.0%, as recommended in the current guidelines (10)). We found that subsequent incident CV events were significantly more frequent among patients with both hs-TnI levels > 4.10 pg/mL and hemoglobin A1C > 7.0% (20.6%) (*p* = 0.018) (Figure 1B). The survival analysis using the Kaplan-Meier curve was shown in Figure 2.

### 3.4. ROC-AUC Determination of Urine hs-TnI Cutoff Values for Association with Subsequent Incident CV Events

**Figure 3:**
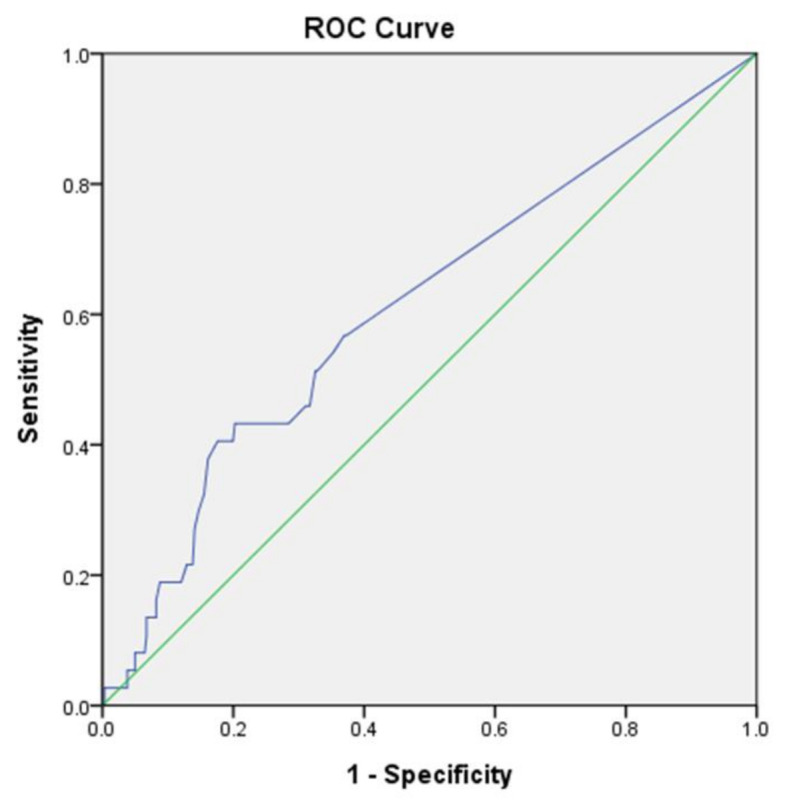
Receiver-operating characteristic curve analysis of fresh urine hs-TnI concentration vs. incident cardiovascular events in patients with DM. Urine hs-TnI concentration (pg/mL): Cut-off value, 1.55; sensitivity, 56.8%; specificity, 63.0%; AUC, 0.611; 95% CI, 0.511–0.711; *p* = 0.027.

We found that the optimal urine hs-TnI cutoff value for association with incident CV events within 3 months was 1.55 pg/mL (sensitivity, 56.8%; specificity, 63.0%; AUC, 0.611; 95% CI, 0.511–0.711; *p* = 0.027) (Figure 3). 

## 4. Discussion

Among patients with DM, we found that urine hs-TnI > 4.10 pg/mL was independently associated with subsequent incident CV events within 3 months. Therefore, a single, non-invasive and skill-independent test of fresh urine provides an accurate measure of the hs-TnI level and may prove useful in the clinical setting for predicting short-term subsequent incident CV events in patients with DM.

DM is a leading cause of death worldwide, much of which is attributed to cardiovascular disease, including acute coronary syndrome and heart failure [11]. The cardiac troponins TnT and TnI are key biomarkers used for the diagnosis and prognosis of acute coronary syndrome [3]. A community-based population study reports that chronic hyperglycemia contributes to myocardial injury beyond its effects on the development of clinical atherosclerotic coronary disease, as assessed by elevated serum levels of hs-TnT [12]. Compared with serum hs-TnT, hs-TnI is shown to be a more sensitive assay for subtle myocardial damage and is significantly detectable in the general population [13]. The early detection of subtle myocardial injury or necrosis as detected by serum hs-TnI has been proved to be an independent predictor for increasing cardiovascular-related long-term outcomes in stable DM patients [5]. Moreover, the serial assessment of hs-TnI has been shown to improve risk stratification of patients with DM [6]. One study showed that the presence of the cardiac TnT detected using a targeted mass spectrometry assay can be demonstrated in the urine of patients with acute myocardial infarction [14]. Several studies have documented the presence and detectability of TnI in urine [8,9,15], providing a non-invasive means of hs-TnI measurement which is more suitable than blood tests for use in outpatient or community-screening settings. Here, we prospectively assessed urine hs-TnI in patients with DM in an outpatient setting to investigate its power for predicting incident CV events during the subsequent 3 months.

Our study population is at high risk for subsequent CV events since more patients have a previous history of hypertension (72.8%), CKD (29.6%), CAD (43.9%), and heart failure with reduced (16.1%) or preserved (17.5%) ejection fraction. Therefore, our observation that 9.8% of the patients had subsequent incident CV events, most related to heart failure, is not surprising. One study reports that the elevated serum hs-TnI (male, 8.5 pg/mL; female, 7.6 pg/mL) is independently associated with increased risk for a major cardiovascular event, heart failure, and myocardial infarction in stable patients with type 2 DM over the course of a 4-year follow up [5]. Our study consistently shows that the short-term incidence of CV events is elevated in patients with DM with urine hs-TnI > 4.10 pg/mL. In our outpatient setting, the urine measurement is more convenient and less invasive than blood sampling. Compared to the previous study showing limited sensitivity (62.7%) and a poor positive predictive value (38.5%) of serum hs-TnI [5], our results showed that urine hs-TnI has limited sensitivity (56.8%) using the cutoff value of 1.55 pg/mL. However, the observed excellent negative predictive value (92.2%) is very promising for the clinical application of this test to managing patients with DM.

Our results also show that the occurrence of micro- and macro-albuminuria did not differ significantly between patients with and without incident CV events, although the UACR was shown to be associated with CKD and predictive of subsequent CV events in a general Japanese population [16] and in patients with type 2 DM [17]. UACR was also shown to be independently associated with increased risk of a wide spectrum of adverse cardiovascular outcomes in patients with type 2 DM. However, the prognostic value of UACR was minimal when evaluated together with other cardiac biomarkers such as hs-TnT, hsCRP, and NT-proBNP [18]. In contrast, we found that UACR correlates significantly with urine TnI in patients with CKD (r = 0.252; *p* = 0.009) but not in those without CKD (r = 0.003; *p* = 0.967). In patients with DM and CKD, a significantly higher urine hs-TnI was found in those positive for microalbuminuria (UACR > 30) (3.78 ± 6.02 vs. 2.00 ± 2.78 pg/mL; *p* = 0.041) and positive for macroalbuminuria (UACR > 300) (6.51 ± 8.50 vs. 1.96 ± 2.45 pg/mL; *p* < 0.012). RAAS blockers (commonly, angiotensin-converting enzyme inhibitors and angiotensin-receptor blockers) can effectively improve micro/macroproteinuria, slow renal disease progression, and prevent the development of cardiovascular disease [19]. Accordingly, guideline-derived medical therapy was common in our study cohort and did not differ significantly between those with and without subsequent incident CV events (Table 1). 

Our multivariate logistic regression analysis indicated that the urine hs-TnI level > 4.10 pg/mL was an independent predictor of subsequent incident CV events, even after adjustment for age, sex, eGFR, hemoglobin A1C > 7.0%, and a previous history of heart failure with reduced ejection fraction (Table 2). This result suggests that in patients with DM, the urine hs-TnI level is better for the early detection of myocardial damage than are the well-known markers of eGFR, UACR, and hemoglobin A1C.

### Limitations

This study has several limitations. First, the sample size was relatively small. In addition, the short-term outcome (incident CV events within 3 months) may not be applicable to long-term outcomes. Second, urinary hs-TnI was measured only once, so we cannot exclude intra-patient sampling variability. Third, urine sampling was obtained during two time periods, which may have affected the results. The average difference between the measurements taken during the two time periods is less than 5% of the values. While sampling of the first urine after waking up in the morning or a 24-h urine collection may be most accurate, such a sampling is not practical for outpatient use. Fourth, the absence of serum hs-TnI data from the patients in our study cohort precluded the comparison between urine and serum hs-TnI. Fifth, urine hs-TnI could not be normalized by urine creatinine since 60.8% patients had the urine hs-TnI level of 0.75 pg/mL due to the limit of detection. Finally, while the AUC of the ROC for urine hs-TnI may be not excellent, it may be a suitable marker for clinical physicians to improve the care of DM patients. A further intervention study using our cutoff value for hs-TnI is warranted. 

## 5. Conclusions

A single measurement of hs-TnI in fresh urine may be a clinically useful marker for predicting incident CV events during the following 3 months in patients with DM. This non-invasive, simple assessment may be a useful tool in clinical settings for monitoring subsequent cardiovascular outcomes in patients with DM.

## Figures and Tables

**Table 1:** Baseline characteristics of the patient cohort.

	Total (*n* = 378)	Incident CV Events (−) (*n* = 341)	Incident CV Events (+) (*n* = 37)	*p*
Age (years)	68.1 ± 11.0	68.1 ± 11.1	67.9 ± 10.0	0.919
Male sex (*n*)	248 (65.6%)	225 (66.0%)	23 (62.2%)	0.642
Body height (cm)	162.0 ± 8.8	162.0 ± 8.8	162.2 ± 8.2	0.889
Body weight (kg)	69.4 ± 13.1	69.7 ± 13.3	66.5 ± 10.9	0.163
Body mass index (kg/m^2^)	26.4 ± 4.0	26.5 ± 4.0	25.8 ± 3.6	0.180
Systolic blood pressure (mmHg)	127 ± 16	127.5 ± 15.7	124.8 ± 17.8	0.197
Diastolic blood pressure (mmHg)	74 ± 10	74.0 ± 10.1	71.8 ± 9.4	0.212
Heart rate (bpm)	80 ± 13	80 ± 14	82 ± 13	0.475
DM duration (years)	7.6 ± 5.5	7.7 ± 5.6	6.8 ± 4.4	0.385
Smoking history (*n*, %)	16 (4.2%)	14 (4.1%)	2 (5.4%)	0.601
Hypertension (*n*)	275 (72.8%)	252 (73.9%)	23 (62.2%)	0.128
Dyslipidemia (*n*)	225 (59.5%)	207 (60.7%)	18 (48.6%)	0.156
Coronary artery disease (*n*)	166 (43.9%)	146 (42.8%)	20 (54.1%)	0.191
Heart failure ejection fraction > 50% (*n*, %)	61 (16.1%)	53 (15.5%)	8 (13.1%)	0.340
Heart failure ejection fraction < 50% (*n*, %)	66 (17.5%)	52 (15.2%)	14 (37.8%)	**0.001**
Atrial fibrillation (*n*, %)	94 (24.9%)	84 (24.6%)	10 (27.0%)	0.749
Chronic kidney disease (*n*, %)	112 (29.6%)	96 (28.2%)	16 (43.2%)	0.056
Creatinine (mg/dL)	1.15 ± 1.02	1.12 ± 0.97	1.43 ± 1.40	0.197
eGFR (mL/min/1.73 m^2^)	70.0 ± 23.0	70.8 ± 22.8	62.1 ± 24.1	**0.029**
Sodium (meq/L)	141 ± 25	139 ± 3	145 ± 4	0.349
Potassium (meq/L)	4.3 ± 0.5	4.3 ± 0.4	4.1 ± 0.5	0.288
Alanine aminotransferase (mg/dL)	29 ± 22	28 ± 19	36 ± 40	0.247
Low-density lipoprotein (mg/dL)	92 ± 31	93 ± 31	88 ± 33	0.506
High-density lipoprotein (mg/dL)	49 ± 21	49 ± 22	45 ± 14	0.427
Triglyceride (mg/dL)	138 ± 84	139 ± 86	132 ± 67	0.718
NT-proBNP (pg/mL) in patients with heart failure ejection fraction < 50%	3400 ± 4978	2245 ± 2565	6483 ± 8005	0.097
Hemoglobin (mg/dL)	12.8 ± 2.1	12.9 ± 2.1	12.4 ± 1.6	0.178
Hemoglobin A1C (%)	7.4 ± 1.3	7.4 ± 1.3	7.5 ± 1.5	0.668
Urine creatinine (mg/dL)	78.4 ± 58.7	79.5 ± 59.9	68.5 ± 47.2	0.280
Urine microalbumin (mg/dL)	27.0 ± 186.4	27.8 ± 195.9	20.0 ± 49.8	0.810
Urine albumin/creatinine ratio (mg/g Cr)	413.9 ± 3232.5	429.2 ± 3400.2	277.6 ± 724.8	0.787
Urine high-sensitivity troponin I (pg/mL)	2.80 ± 4.22	2.65 ± 4.03	4.18 ± 5.59	**0.036**
Urine high-sensitivity troponin I/creatinine ratio (pg/mg Cr)	7.51 ± 16.56	7.25 ± 16.67	9.88 ± 15.62	0.339
Urine high-sensitivity troponin I > 4.10 (pg/mL)	75 (19.8%)	60 (17.6%)	15 (40.5%)	**0.001**
**Medications taken (*n*, patients; %)**				
Antiplatelet	203 (53.7%)	181 (53.1%)	22 (59.5%)	0.460
ACEI	46 (12.2%)	42 (12.3%)	4 (10.8%)	0.790
ARB	194 (51.3%)	178 (52.2%)	16 (43.2%)	0.301
ARNI	16 (4.2%)	14 (4.1%)	2 (5.4%)	0.663
MRA	28 (7.4%)	24 (7.0%)	4 (10.8%)	0.720
Beta blockers	206 (54.5%)	184 (54.0%)	22 (59.5%)	0.523
Diuretics	70 (18.5%)	62 (18.2%)	8 (21.6%)	0.609
Statins	266 (70.4%)	240 (70.4%)	26 (70.3%)	0.989
Biguanide	199 (52.6%)	181 (53.1%)	18 (48.6%)	0.608
Dipeptidyl peptidase 4 inhibitor	96 (25.4%)	88 (25.8%)	8 (21.6%)	0.579
Sulfonylurea	67 (17.7%)	61 (17.9%)	6 (16.2%)	0.800
*α*-*Glucosidase* inhibitor	7 (1.9%)	6 (1.8%)	1 (2.7%)	0.517
Thiazolidinedione	4 (1.1%)	3 (0.9%)	1 (2.7%)	0.339
Meglitinide	39 (10.3%)	36 (10.6%)	3 (8.1%)	1.000
Sodium glucose co-transporters 2 inhibitor	56 (14.8%)	48 (14.1%)	8 (21.6%)	0.220
Insulin	48 (12.7%)	42 (12.3%)	6 (16.2%)	0.445

Data are presented as the mean ± standard deviation. ACEI: Angiotensin converting enzyme inhibitor; ARB: Angiotensin II receptor blocker; ARNI: Angiotensin receptor neprilysin inhibitor; CV: Cardiovascular; DM: Diabetes mellitus, eGFR: Estimated glomerular filtration rate; MRA: Mineralocorticoid receptor antagonist; NT-proBNP: N-terminal pro b-type natriuretic peptide.

**Table 2:** Multivariate logistic regression analysis of independent predictors for subsequent incident CV events in patients with DM.

	OR	95% CI for B	*p*
Age (years)	0.992	0.960–1.025	0.617
Sex (male)	0.757	0.361–1.591	0.463
Creatinine (mg/dL)	1.155	0.911–1.465	0.234
Heart failure ejection fraction < 50% (yes)	3.051	1.442–6.458	0.004
Urine hs-TnI > 4.10 (pg/mL) (yes)	2.762	1.322–5.769	0.007
Age (years)	0.982	0.947–1.017	0.312
Sex (male)	0.771	0.367–1.620	0.492
Estimated glomerular filtration rate (mL/min/1.73 m^2^)	0.986	0.972–1.001	0.074
Heart failure ejection fraction < 50% (yes)	2.738	1.279–5.859	0.009
Urine hs-TnI > 4.10 (pg/mL) (yes)	2.880	1.383–5.995	0.005
Age (years)	0.979	0.944–1.016	0.260
Sex (male)	0.780	0.363–1.675	0.524
Estimated glomerular filtration rate (mL/min/1.73 m^2^)	0.987	0.971–1.002	0.096
Hemoglobin A1C > 7.0% (yes)	1.402	0.676–2.908	0.364
Heart failure ejection fraction < 50% (yes)	2.855	1.319–6.180	0.008
Urine hs-TnI > 4.10 (pg/mL) (yes)	3.115	1.478–6.563	0.003

hs-TnI: High-sensitive troponin I.

## Abbreviations

ACS	Acute coronary syndrome
AUC	Area under the curve
BMI	Body mass index
CKD	Chronic kidney disease
CV	Cardiovascular
DM	Diabetes mellitus
eGFR	Estimated glomerular filtration rate
hs-TnI	High-sensitivity cardiac troponin I
RAAS	*Renin-angiotensin-aldosterone system*
ROC	Receiver-operating characteristic
SGLT_2_	Sodium glucose co-transporters 2
UACR	Urine albumin-creatinine ratio.
